# Right Breast Mastectomy and Reconstruction with Tissue Expander under Thoracic Paravertebral Blocks in a 12-Week Parturient

**DOI:** 10.1155/2015/842725

**Published:** 2015-07-02

**Authors:** Christopher Allen-John Webb, Paul David Weyker, Shara Cohn, Amanda Wheeler, Jennifer Lee

**Affiliations:** ^1^Department of Anesthesiology, Perioperative & Pain Medicine, Stanford University School of Medicine, Stanford, CA, USA; ^2^Department of Anesthesia & Perioperative Care, San Francisco School of Medicine, University of California, San Francisco, CA, USA; ^3^Department of Surgery, Stanford University School of Medicine, Stanford, CA, USA

## Abstract

Paravertebral blocks are becoming increasingly utilized for breast surgery with studies showing improved postoperative pain control, decreased need for opioids, and less nausea and vomiting. We describe the anesthetic management of an otherwise healthy woman who was 12 weeks pregnant presenting for treatment of her breast cancer. For patients undergoing breast mastectomy and reconstruction with tissue expanders, paravertebral blocks offer an anesthetic alternative when general anesthesia is not desired.

## 1. Introduction 

Paravertebral blocks are becoming increasingly utilized for breast surgery with studies showing improved postoperative pain control, decreased need for opioids, and less nausea and vomiting [1, 2]. We describe the anesthetic management of an otherwise healthy woman who was 12 weeks pregnant presenting for right breast mastectomy and reconstruction with tissue expander for treatment of her breast cancer. Given her concerns of anesthesia during the first trimester, she wished to proceed without general anesthesia.

## 2. Case Description

**Figure 1:**
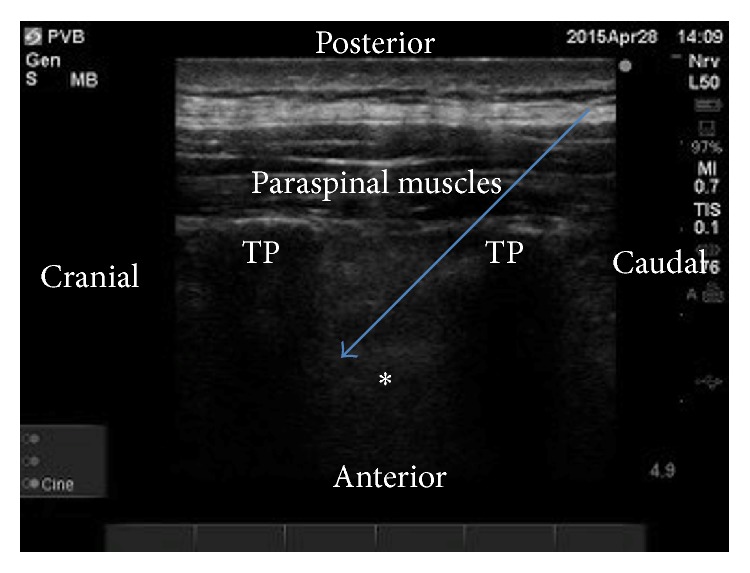
Using a linear transducer (10–5 MHz, Sonosite Bothel, WA), a paramedian oblique sagittal scan was used to obtain the view of the transverse processes. A slight lateral tilt was applied to optimize visualization of the pleural lining (white asterisk). The needle was advanced in a caudal to cranial direction until the tip was placed underneath the costotransverse ligament. After negative aspiration, hydrodissection with saline was used and anterior displacement of the pleura was confirmed. TP = transverse process. Blue arrow delineates needle trajectory. The white asterisk represents the pleura.

With approval from the Stanford University IRB and waiver of consent, we reviewed the record of a 40-year-old (55 kilogram) parturient who underwent a right breast nipple sparing mastectomy and reconstruction with tissue expanders under paravertebral blocks. At 9 weeks of gestation, she underwent a right breast lumpectomy and sentinel lymph node dissection with infiltration of local anesthetic and monitored anesthesia care. Given the presence of extensive multifocal lymphovascular invasion and positive medial margins combined with her desire to postpone chemotherapy until after the first trimester, it was recommended that she proceed with a complete mastectomy. On the day of surgery we discussed the possibility of performing multiple level paravertebral blocks with or without intraoperative sedation versus a high thoracic epidural, or general anesthesia. After discussions with her maternal fetal medicine physician and her surgeon, she decided to undergo the paravertebral blocks as her primary anesthetic. While paravertebral catheters were offered, she decided that the combination of single injection blocks with nonopioid analgesics would be her therapy of choice for postoperative pain control. After obtaining informed consent and completing a formal time out, the patient was placed in the prone position and standard American Society of Anesthesiology (ASA) monitors were attached. In order to provide surgical anesthesia, we decided to perform three, single injections of local anesthetic at the second, forth, and sixth thoracic (T) levels [3]. Using a linear transducer (10–5 MHz, Sonosite Bothel, WA), the probe was placed over the right costal margins in a longitudinal axis to produce a sagittal view of the ribs and intercostal spaces. The ribs were then counted and the second, forth, and sixth ribs were marked to delineate the T2, T4, and T6 levels. The skin was then cleaned with a 2% chlorhexidine gluconate and 70% isopropyl alcohol solution sterile towels were placed on the patient. A sterile probe cover was also applied to the transducer. Using an in-plane, paramedian oblique sagittal technique [4], the right T2 and T3 transverse processes were identified (Figure 1). A slight lateral tilt was applied to better delineate the pleura and the superior costotransverse ligament. The probe was then moved cephalad until the T3 transverse process was at the edge of the ultrasound screen. A 20-gauge, 100 millimeter echogenic stimulating needle (Pajunk Norcross, GA) was advanced in-plane under direct visualization. The tip was advanced through the costotransverse ligament. After negative aspiration, hydrodissection with saline was used and anterior displacement of the pleura was confirmed. Ten milliliters (mL) of 0.5% ropivacaine was then injected in 2-3 mL increments with intermittent negative aspiration. Anterior displacement of the pleura was again visualized. The same procedure was then performed on the right side at the T4 and T6 levels. Prior to proceeding to the operating room, dermatomal levels were checked (to pinprick sensation) and confirmed an appropriate band of anesthesia over the right T1–T10 dermatome levels. Intraoperatively, she received sedation with a Propofol infusion at 50–80 micrograms/kilogram/minute in addition to intermittent boluses of intravenous fentanyl (total 200 micrograms) to provide additional analgesia.

Postoperatively, she reported zero pain overnight and did not require any medications for analgesia. On the morning of postoperative day (POD), one, approximately 17 hours after the injections, she received two tablets of Oxycodone/Acetaminophen 5/325 for a pain score of 5. Additionally, she also received two tablets of acetaminophen 500 milligrams. By the end of POD 1, she had complete return of sensation of her anterior chest wall. She did not report any complaints of nausea or vomiting during her hospital stay. She was discharged from the hospital on POD 2 with a pain score of 3 and did not require any additional analgesics.

## 3. Discussion

Pregnant patients presenting for surgery during the first trimester often present a difficult challenge to the anesthesiologist. While there has been no definitive correlation to increased risk of congenital anomalies in pregnant women who undergo anesthesia during pregnancy, there is an increased risk for spontaneous abortion and low birth weight when anesthesia exposure occurs during the first trimester [5]. Nitrous oxide has been shown to impair DNA synthesis; however, this has not been shown to have any clinical implications. The currently available volatile anesthetics (isoflurane, sevoflurane, and desflurane) as well as the common induction agents (Propofol, etomidate, and ketamine) are considered to be safe during pregnancy [5]. Similarly, opioids and acetaminophen are category B medications and are considered to be safe during pregnancy [6]. However, the long-term effects of maternal exposure to anesthetics on neurodevelopment are still not currently known [7]. Changes in maternal physiology also affect drug metabolism and thus the pharmacokinetics of certain medications will be altered [8]. Decreased concentrations of albumin and alpha-1-acid glycoprotein (AAG) result in a lower volume of distribution and total body clearance of local anesthetics [8, 9].

For patients presenting for breast mastectomy and reconstruction with tissue expanders, paravertebral blocks offer an anesthetic alternative when general anesthesia is not desired. Similar to high thoracic epidurals or cervical epidurals, paravertebral blocks are able to provide surgical anesthesia for procedures involving the anterior chest wall. In our case, the patient did receive additional fentanyl at the beginning of the case. This was expected as cutaneous innervation oftentimes takes longer to become anesthetized when using regional techniques. The combination of intravenous analgesics and local infiltration at the incision site can be used to treat pain on incision. On POD one, the timing of her breakthrough pain was about 17 hours after the block was performed. While the duration of analgesia of peripheral nerve blocks following 0.5% ropivacaine is considered to be 12–15 hours, the slightly longer duration may be related to her circulating levels of AAG [10]. By POD two, her pain was entirely managed with nonopioid analgesics. For pregnant patients presenting for mastectomy and breast reconstruction, paravertebral blocks are a safe and reasonable alternative to general anesthesia.
